# Global research trends in endometrial receptivity from 2000 to 2024: bibliometric analysis

**DOI:** 10.3389/fmed.2024.1465893

**Published:** 2024-10-30

**Authors:** Ziping Liu, Zelin Zhang, Ping Xie

**Affiliations:** ^1^College of Clinical Medicine, Chengdu University of Traditional Chinese Medicine, Chengdu, China; ^2^Department of Gynecology, Hospital of Chengdu University of Traditional Chinese Medicine, Chengdu, China

**Keywords:** endometrial receptivity, citespace, VOSviewer, research hotspots, bibliometric analysis

## Abstract

**Background:**

In recent years, extensive research has been conducted on endometrial receptivity (ER), with rapidly evolving research hotspots and trends. Our study aimed to explore the development of ER research from 2000 to the present and provide insights for future endeavors.

**Materials and methods:**

Relevant research publications on ER from 2000 to 2024 were retrieved from the Web of Science Core Collection (WOSCC) database. CiteSpace, VOSviewer and Excel tools were employed to conduct the bibliometric analysis.

**Results:**

A total of 3,354 articles were analyzed, revealing an overall upward trend in annual publication numbers, signifying the increasing attractiveness and research value of this field. Globally, China led with a notable advantage of 1,030 publications, followed by the United States (650) and Spain (251), constituting the first tier of international research. Valencia University topped the list of institutions with 108 publications, closely followed by Shanghai Jiao Tong University with 87. *Fertility and Sterility* (IF6.6, Q1) is the one with the largest number of publications, accounting for 7.96% of the total publications. The three most co-cited journals were *Fertility and Sterility*, *Biology of Reproduction*, and *Human Reproduction*. A co-citation reference analysis revealed that ER research can be categorized into ten major subfields, including embryo implantation, frozen embryo transfer, integrins, recurrent implantation failure, intrauterine adhesions, etc. Since 2020, the keywords with the strongest citation bursts include repeated implantation failure and frozen.

**Conclusion:**

This study employs bibliometric analysis to offer researchers in the field of ER a comprehensive perspective. Since 2000, there has been a remarkable surge in the number of publications in the ER research field. These studies primarily concentrate on delving into the pathophysiological mechanisms of ER, with the primary objective of enhancing clinical pregnancy rates and live birth rates, benefiting more infertile patients. Currently, addressing the ER issues in patients with recurrent implantation failure represents the forefront of research. The primary treatment approaches currently in use involve optimizing embryo transfer timing and employing innovative strategies such as immunotherapy. These cutting-edge analyses not only provide new insights into the treatment of ER but also offer researchers fresh research directions, and staying abreast of the latest trends and advancements in the field.

## 1 Introduction

Endometrial Receptivity (ER), a key determinant of pregnancy success, refers to the ability of the endometrium to accommodate the embryo and complete its localization and implantation during the Window of Implantation (WIO), a subtle stage that is usually located in the mid-to-late luteal phase of the human luteal phase, i.e., days 20 to 24 of the menstrual cycle. The WIO refers specifically to the ability of the uterine lining to accept the embryo and to localize, adhere and implant during the WIO, a delicate stage usually located in the mid-to-late luteal phase of the human menstrual cycle, between days 20 and 24. This characteristic directly maps the endometrium’s receptivity to the embryo and is essential for successful embryo attachment and subsequent development (1). The transient nature of WIO makes it a major challenge in reproductive medicine research, during which the endometrium carefully creates a microenvironment conducive to embryo adhesion, implantation, and growth by secreting a variety of enzymes and cytokines through fine-tuned regulation (2).

However, the intricate interplay among hormones, immune factors, cytokines, and adhesion molecules underpinning this process remains elusive, necessitating profound exploration (3).

According to the World Health Organization’s (WHO) “Infertility Prevalence Estimates (1990–2021)” report, approximately 17.5% of adults worldwide suffer from infertility (4). Although assisted reproductive technology (ART), particularly *in-vitro* fertilization and embryo transfer (IVF-ET), has significantly improved outcomes for infertility caused by tubal dysfunction, pregnancy success rates are still constrained by multiple factors. Studies have highlighted that embryo implantation rates, influenced by both embryo and endometrial factors, are decisive in IVF-ET success (5, 6). Notably, inadequate ER stands out as the primary cause of implantation failure, accounting for up to two-thirds of cases (7). Consequently, enhancing ER has emerged as a crucial strategy to improve clinical pregnancy rates.

In recent years, there has been a surge of research focusing on various indicators for evaluating ER, encompassing a multidimensional assessment system that incorporates ultrasonic markers (such as endometrial thickness, pattern, and volumetric blood flow perfusion), morphological markers (e.g., pinopodes), and molecular biological markers [including leukemia inhibitory factor (LIF), integrin αVβ3, and forkhead box O1 (FoxO1)]. These studies have provided new insights into clinical assessment and intervention strategies (8–10). In order to address the issue of reduced ER, researchers have attempted various methods, including endocrine imbalance correction, immune function regulation, anticoagulant therapy, nutritional supplementation, and adjustment of implantation timing (11–14). Nevertheless, due to the incomplete understanding of the underlying mechanisms of ER, the therapeutic prospects remain limited, resulting in decreased implantation and pregnancy rates.

Bibliometrics, as a powerful tool within the realm of informetrics, exhibits extensive interdisciplinary applications, playing an indispensable role in deepening both theoretical and practical research endeavors. It systematically organizes and quantifies vast amounts of research literature, thereby effectively anticipating the developmental trajectory of disciplines and serving as a cornerstone in knowledge management and information mining. Through this methodology, we can swiftly grasp the panoramic view of ER research, precisely identify research hotspots, and illuminate pathways for further exploration and improvement within the ER domain. Notably, despite its vast potential for application, bibliometrics’ capacity to evaluate the research effectiveness in the ER field remains largely untapped. This underscores the imperative for us to actively explore and promote this methodology in the future. Given this context, the present study innovatively employs VOSviewer and CiteSpace visualization tools and Excel (15, 16) to conduct an in-depth analysis of ER-related literature spanning from 2000 to 2024. Our primary objective is to unravel the dynamic shifts in ER research hotspots within this timeframe and forecast future trends, concurrently identifying potential hotspots that may steer future research directions. This endeavor aims to provide invaluable insights and inspiration to researchers within the field.

## 2 Materials and methods

### 2.1 Search strategy

The data utilized in this study were retrieved and downloaded from WoSCC on June 12, 2024. We employed the following search formula: The search formula was set to [TS = (Endometrial Receptivity”], language (English), Publication Date: (2000-01-01)–(2024-05-31), literature type (Article or Review). After eliminating irrelevant publications, a total of 3,354 articles were identified (excluding duplicates). The retrieved articles were saved in plain text format and exported as complete records, including their cited references. The selection of the time range from 2000 to 2024 as the research window is based on careful consideration of the development dynamics in this field. Since 2000, with the rapid development of medicine, biology, information technology and other fields, the research methods and theoretical framework of ER Research paradigms have undergone significant changes, and ER is a dynamically developing research field, with new research results constantly emerging and old theories and perspectives constantly being revised or replaced. Therefore, in order to focus on the latest research results, technological trends and theoretical frontiers, we selected literature from 2000 to the present as the research basis. The documents exported from WOSCC were imported into Citespace for deduplication. Subsequently, Excel was used to verify that all documents were within that time period. The selected documents were then imported into VOSviewer and CiteSpace for in-depth and comprehensive analysis.

### 2.2 Data analysis

To visually present and analyze the retrieved literature, we employed bibliometric tools such as VOSviewer, CiteSpace, and Excel. To better showcase the achievements in our ER research field, we selected appropriate software to analyze various aspects of ER studies. In this research, we leveraged CiteSpace (version 6.3.R1) and VOSviewer (version 1.6.20) to conduct visual and precise analyses of publications, countries, institutions, authors, cited documents, and keywords from the retrieved literature. Specifically, we utilized VOSviewer (version 1.6.20)^1^ (16), developed by Nees Jan van Eck and Ludo Waltman at Leiden University, to conduct a detailed analysis of publications, countries/regions, publishing institutions, journals, authors, keywords, co-cited journals, co-cited authors, and co-cited documents.

Within VOSviewer, we meticulously set thresholds of a minimum frequency of 200 for cited journals, 150 for cited authors, and 70 for article citation counts, ensuring the representativeness and accuracy of our analytical outcomes. These settings facilitated the identification of the most influential journals, authors, and documents within the field, unveiling their citation relationships and academic network structures.

Furthermore, we employed CiteSpace (version 6.3.R1, a widely used bibliometric analysis and visualization software developed by Professor Chen Chaomei) (15) to conduct a visual analysis of keywords. Through its unique “burst” visualization technique, the software vividly portrayed the evolutionary trajectory and intrinsic connections of research themes. The size and color intensity of the “bursts” corresponded to the frequency of occurrence and temporal distribution of the nodal content, while the connecting lines between nodes revealed the strength of co-occurrence and the closeness of their associations. In terms of parameter settings, we focused on data spanning from 2000 to 2024, adopting a one-year time slicing strategy. We selected “keywords” as the node type. The network structure was optimized using the “Pathfinder” pruning algorithm and the “pruning splited networks” strategy, ultimately presenting the consolidated network in the “cluster view-static” mode, yielding an informative knowledge map.

## 3 Results

### 3.1 Number of publications and citation evolution

**FIGURE 1:**
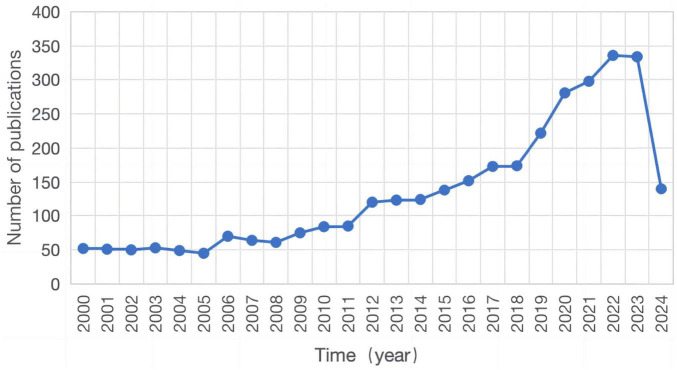
Year of publications.

From the WoSCC database, we retrieved a total of 3,354 publications. A line chart was constructed to illustrate the changes in the number of publications related to endometrial receptivity from 2000 to 2024 (Figure 1). Based on the annual growth rate of publications, the entire period can be divided into three distinct phases: Phase I (2000–2008), characterized as a period of stability; Phase II (2009–2019), marked by a gradual increase; and Phase III (2020–2023), featuring a rapid growth phase.

**FIGURE 2:**
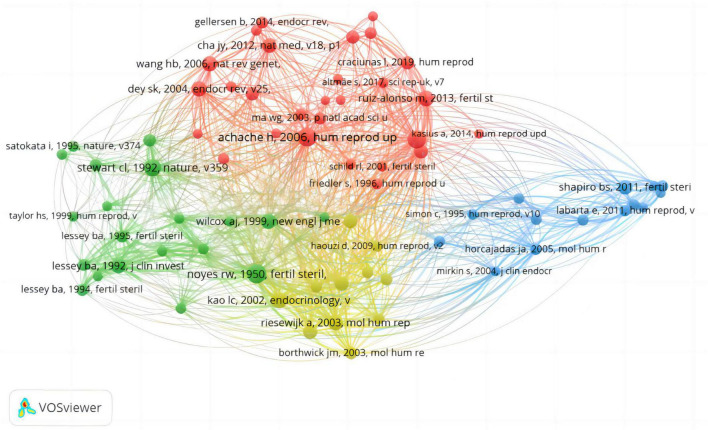
Analysis diagram of co-citation network in publications.

The co-citation network analysis conducted using VOSviewer (depicted in Figure 2) reveals that the most frequently cited article within the WoSCC database is authored by Hanna Achache, titled “*Endometrial receptivity markers, the journey to successful embryo implantation*” (7). Published in 2006 in the *Journal Human Reproduction Update* (Impact Factor = 14.8, Q1), this article has been cited 314 times. The second most cited article is *Dating the Endometrial Biopsy* by Noyes et al. (17), initially published in 1950 and reprinted in *Fertility and Sterility* in 2019 (IF = 6.6, Q1). Following closely in the third position is “*A genomic diagnostic tool for human endometrial receptivity based on the transcriptomic signature*” by Díaz-Gimeno et al. (18), published in 2011 in *Fertility and Sterility* (IF = 6.6,Q1). Through a systematic assessment of citation counts, we analyzed the top five cited articles, all of which report on early endometrial receptivity markers and related gene expression profiles (19, 20). This analysis underscores the significance and impact of these publications in advancing the understanding of ER.

### 3.2 Core journal analysis

**FIGURE 3:**
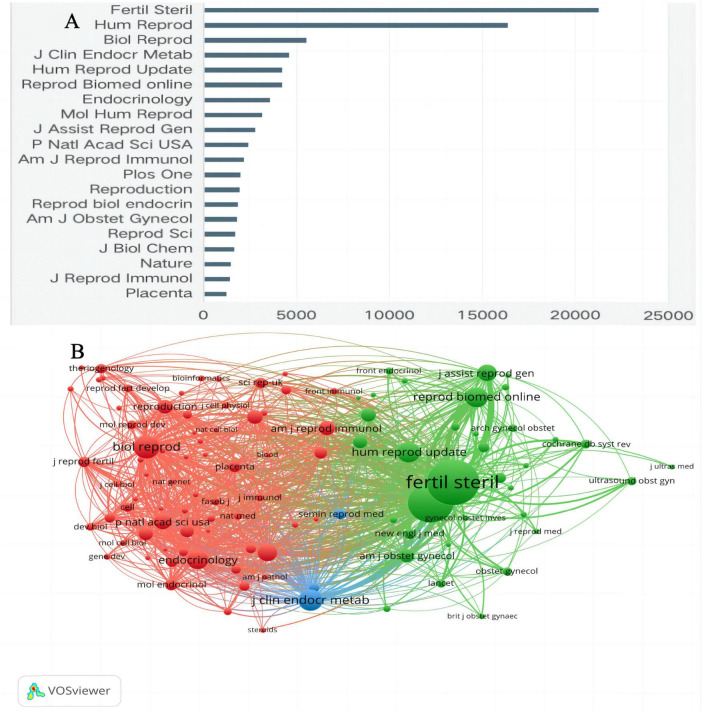
**(A)** The top 20 journals with the highest publication volume on ER and **(B)** Collaborating network of journals on ER.

Figure 3A presents the top 20 journals that have published articles related to ER from 2000 to 2024. Collectively, these journals have contributed 1,611 articles, accounting for 34.61% of the total publications. Notably, *Fertility and Sterility*, with an IF of 6.6 and a Q1 ranking in the Journal Citation Reports, has published 267 relevant articles, constituting 7.96% of all publications. Additionally, the journal co-citation network visualized by VOSviewer (Figure 3B) reveals that the three key journals with the highest overall link strength are *Fertility and Sterility(*IF = 6.6, Q1), *Biology of Reproduction* (abbreviated as *Biol Reprod*, IF = 3.1,Q2), and *Human Reproduction* (abbreviated as *Hum Reprod*, IF = 6.0,Q1). This analysis underscores the significant role these journals play in disseminating research on ER.

### 3.3 National and institutional analysis

**FIGURE 4:**
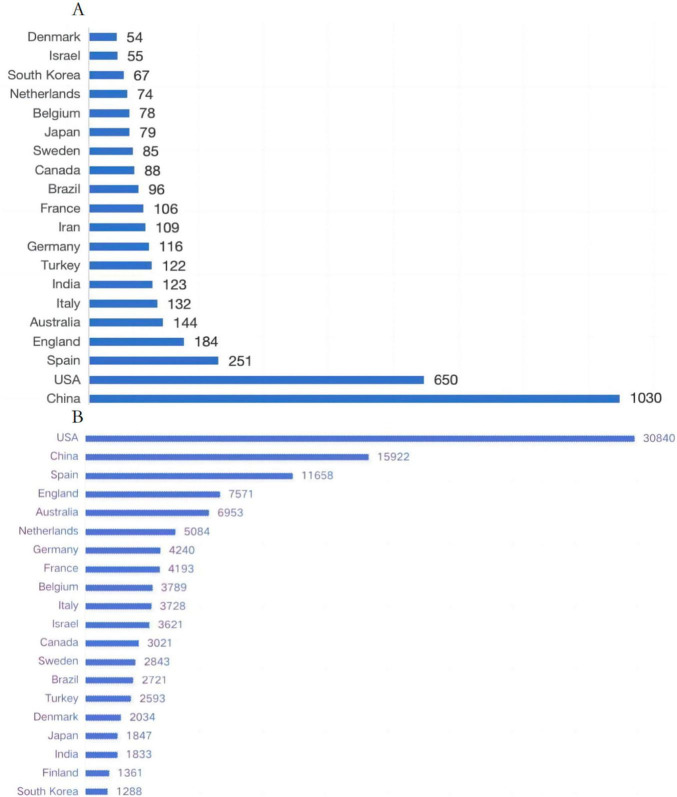
**(A)** The top 20 countries or regions with the highest publication volume on ER and **(B)** The top 20 countries or regions with the highest citation of ER.

**FIGURE 5:**
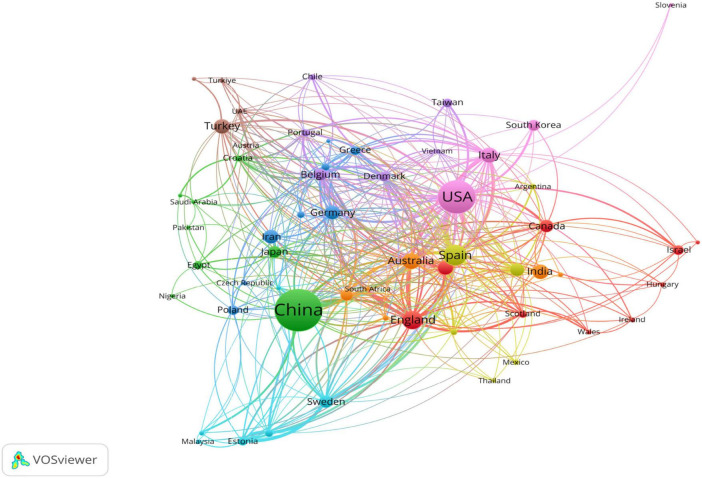
Country or regional co-operation map, which includes at least 5 publications based on cooperation between countries.

Figure 4A presents the number of published articles and the collaborative network among 58 countries in the field of ER research. China leads in productivity with 1,030 articles, followed by the United States (650 articles), Spain (251 articles), Italy (184 articles), and Australia (144 articles). In terms of citation impact, the United States emerges as a significant contributor to the field, with a total of 30,840 citations, followed by China (15,922 citations), Spain (11,658 citations), and other countries. These nations demonstrate a heightened interest in ER research (Figure 4B). Figure 5 further illustrates the collaborative network among these 58 countries, depicting collaborations where each country has contributed at least 5 articles.

**FIGURE 6:**
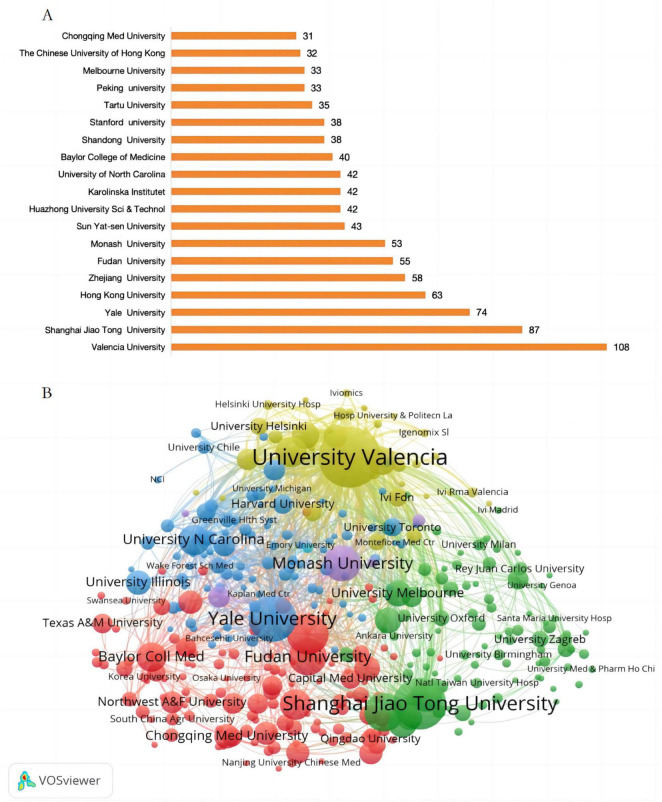
**(A)** The top 20 institutions with the highest production in the field of ER and **(B)** Collaboration network among various institutions on ER.

Figure 6A showcases the top 20 institutions with the highest output in the field of ER research, with eight located in China. The University of Valencia leads with 108 publications, followed closely by Shanghai Jiao Tong University with 87 publications, and Yale University with 74 publications. Figure 6B illustrates the collaboration network among these institutions.

### 3.4 Author contributions

**FIGURE 7:**
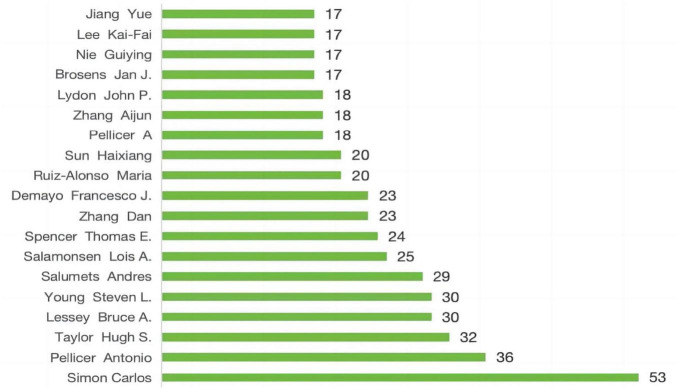
Top 20 authors with the highest number of authors.

**FIGURE 8:**
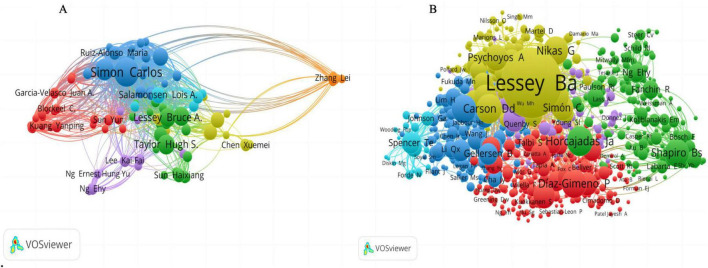
**(A)** Collaborative network of authors participating in at least 10 articles and **(B)** Author’s Co-citation Network.

The top 20 most prolific authors collectively contributed 484 articles, accounting for 14.43% of all published works in this field (Figure 7). Among them, Simon Carlos stands out as the most productive, with 53 publications in this domain, followed by Pellicer Antonio with 36 publications and Taylor Hugh S. with 32 publications. Utilizing VOSviewer, we constructed a collaboration network (Figure 8A) to visualize the interactions among these authors. This network focuses on the collaborations among the 103 authors who have contributed to at least 10 articles, highlighting the close cooperation among them. Notably, Simon Carlos exhibits the most prominent collaboration, with a link strength of 2,404. Subsequently, Pellicer Antonio and Díaz-Gimeno Patricia, with link strengths of 1,409 and 1,157 respectively, also emerge as notable collaborators within this network.

The co-citation network analysis delves deeper into the collaborative relationships among the 80 authors who have been cited in at least 150 articles (Figure 8B). This analysis further underscores the preeminence of Lessey BA, Shapiro BS, and Noyes RW as the top three authors in this context. The co-citation network highlights their significant contributions and the extent to which their work has been integrated and referenced by the broader research community.

### 3.5 Keyword and hotspot analysis

Keywords, as concise and highly generalized representations of research content, encapsulate the core themes and primary ideas of scientific literature. By conducting a co-occurrence analysis of the major keywords found within publications within a specific research domain, researchers can gain an intuitive and comprehensive understanding of the primary research hotspots in that field over recent years. In this analysis, each node represents a unique keyword, with the size of the node reflecting the frequency of its appearance in scientific literature within the domain. The color of the keyword nodes indicates the year of their first occurrence, while the connecting lines between keywords signify their co-occurrence within the same document.

**FIGURE 9:**
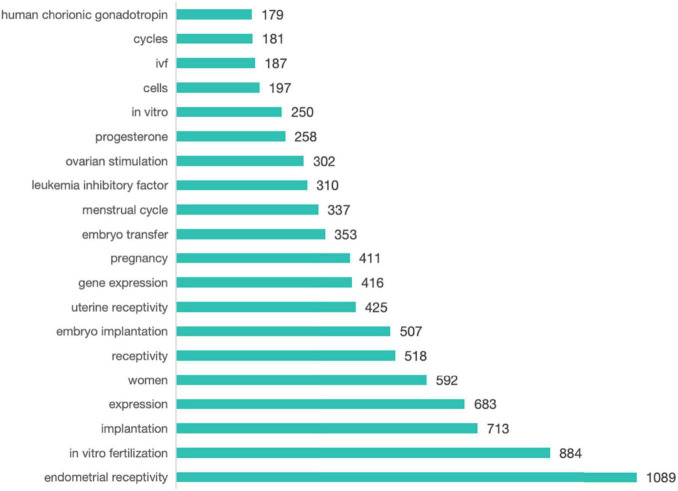
Top20 keyword frequence rankings.

**FIGURE 10:**
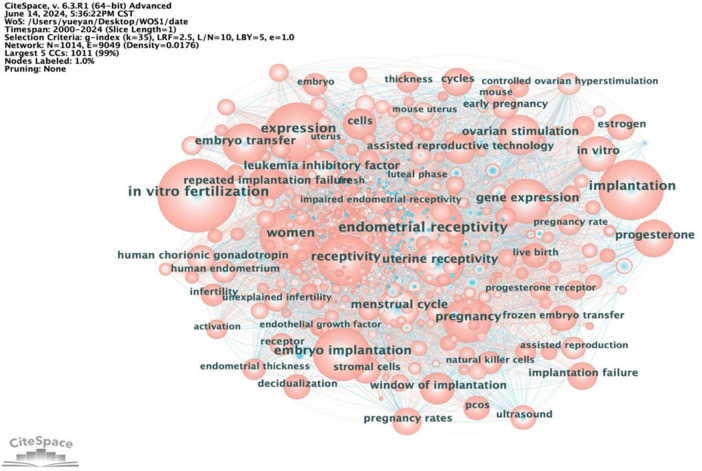
Keyword co-occurrence analysis chart on ER.

Employing Citespace, we conducted a comprehensive keyword analysis of 3,354 articles related to ER. The top 20 keywords ranked by frequency are presented in Figure 9. The keyword co-occurrence map comprises 1,014 nodes and 9,049 links, resulting in a network density of 0.0176, as depicted in Figure 10. Within this network, the betweenness centrality (CB) of a keyword signifies its importance. Keywords with high centrality (CB ≥ 0.1) hold significant value, representing hot topics in the field that garner substantial attention, depth of research, and broad influence (16). In this analysis, the betweenness centrality of the co-occurring keywords was relatively low, with the top five being controlled ovarian hyperstimulation (0.06), cycles (0.05), assisted reproduction (0.05), uterine receptivity (0.04), and human chorionic gonadotropin (0.04).

**FIGURE 11:**
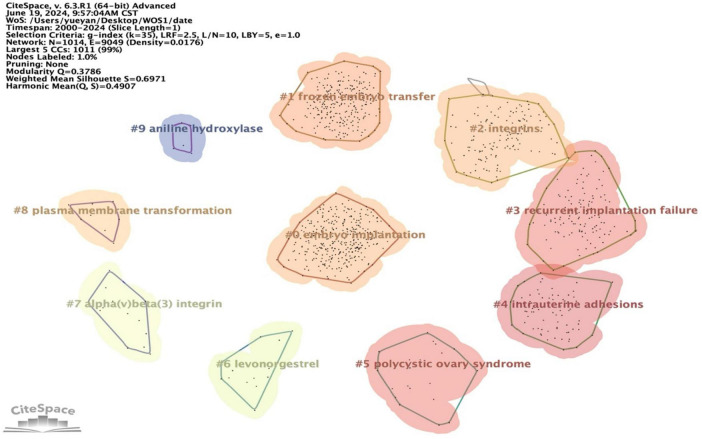
Keyword cluster analysis on ER.

Utilizing CiteSpace, we constructed a clustering map of ER (Figure 11), where distinct clusters are represented by various colors such as red, green, blue, and yellow. Nodes within a cluster sharing the same color signify closely related co-occurrences, with node sizes and link widths varying according to the degree and intensity of these co-occurrences. To evaluate the quality of the resulting cluster network, two indicators were employed: Modularity Q (*Q*-value) and Mean Silhouette (*S*-value). A *Q*-value greater than 0.3 indicates a statistically significant cluster structure, whereas an S value exceeding 0.7 signifies an efficient and convincing clustering process (15). These keyword clusters were subsequently categorized into ten distinct groups, encompassing embryo implantation, frozen embryo transfer, integrins, recurrent implantation failure, intrauterine adhesions, poly plasma membrane, and aniline hydroxylase, among others. Supplementary Table 1 provides specific information for each keyword cluster.

**FIGURE 12:**
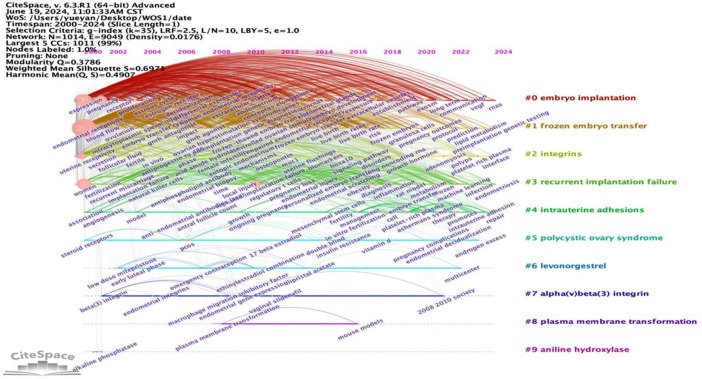
Keyword clustering timeline analysis on ER.

**FIGURE 13:**
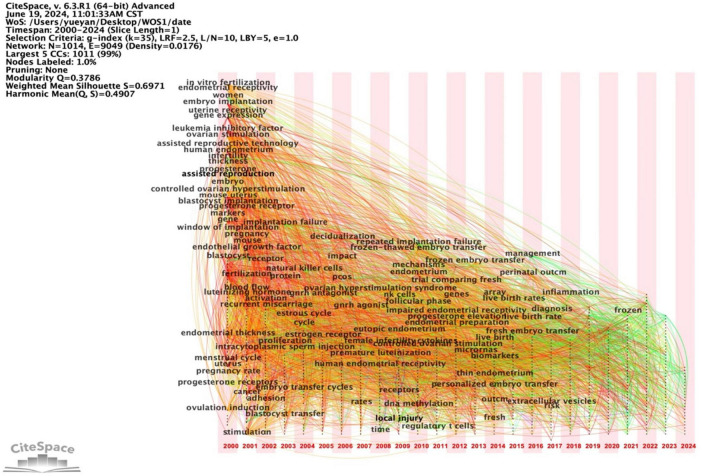
Keyword clustering time zone analysis on ER.

In the literature, the three cluster labels #0 embryo implantation, #1 frozen embryo transfer, and #2 integrins have spanned the entire data collection period, underscoring their significance as primary research themes within the field of ER. Additionally, clusters with broader temporal coverage include #0 embryo implantation, #1 frozen embryo transfer, #2 integrins, and #3 recurrent implantation failure. Notably, high-frequency and high-betweenness centrality keywords predominantly emerged during the early stages of research, such as *in-vitro* fertilization, endometrial receptivity, and embryo implantation, which were present as early as 2000–2004. Between 2005 and 2009, new keywords like frozen embryo transfer, decidualization, local injury, pcos, and dna methylation emerged. From 2010 to 2015, traditional Chinese medicine-related keywords such as impaired ER and live birth rates appeared. More recently, from 2016 to 2024, emerging keywords like inflammation and extracellular vesicles have gained prominence. These trends are illustrated in Figures 12, 13.

In reviewing the literature, the keyword “menstrual cycle” emerged prominently across the period from 2000 to 2013, reflecting the intense focus on studies correlating ER with the menstrual cycle during this timeframe. Concurrently, keywords such as “uterine receptivity,” “estrogen receptor”, “progesterone receptors,” “ultrasound markers”, and “oocyte donation” emerged, highlighting the multifaceted approaches researchers adopted to investigate ER between 2000 and 2010. These approaches included observations of endometrial hormone receptors, color Doppler ultrasound monitoring, endometrial gene expression, studies on the estrous cycle, and explorations of the correlation between oocyte donation and endometrial receptivity.

**FIGURE 14:**
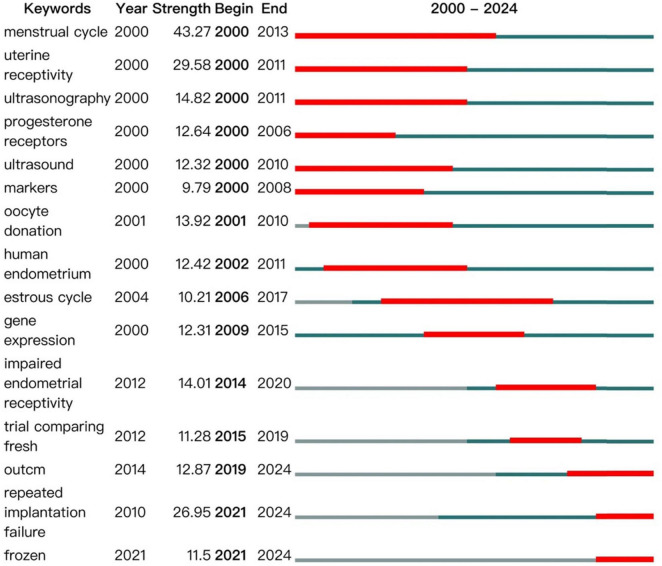
Key word highlights in the field of ER.

From 2012 onwards, particularly intensifying in 2014 and 2015, the research focus shifted towards “impaired endometrial receptivity,” with the emergence of terms like “trials comparing fresh” (likely referring to trials comparing fresh embryo transfers), indicating a new trajectory in research that specifically addressed the issue of compromised endometrial receptivity. Since 2020, the persistent emergence of keywords like “repeated implantation failure” and “frozen” signals that recurrent implantation challenges and the implantation of frozen embryos have emerged as enduring research priorities within this field. The detailed trends are depicted in Figure 14.

In this study, we employed bibliometric tools to analyze ER research from the several perspectives: (1) identifying and quantifying general information to assess individual impact and collaboration dynamics; (2) conducting co-citation analysis to map the intellectual landscape of the knowledge domain; (3) analyzing keywords to discern emerging trends. Our findings provide a robust foundation for exploring the knowledge base within the field of ER.

## 4 Discussion

### 4.1 General information

To gain insights into the focal points and developmental trajectory of ER, we conducted a bibliometric and visualization analysis. The annual output of research products exhibited notable variations. Based on the WoSCC database, from 2000 to May 31, 2024, 13,449 authors from 3,153 institutions across 89 countries/regions contributed 3,354 relevant articles across 583 academic journals. Our research reveals a sustained surge in ER research over the past two decades, particularly within the last five years, marked by a dramatic increase in publications, underscoring the growing attractiveness and research value of this field.

Globally, China leads with a prominent advantage of 1,030 publications, followed by the United States (650) and Spain (251), forming the first tier of international research. Additionally, over 11 countries/regions have contributed more than 100 articles in English, demonstrating the internationalization and collaborative trends within this field. At the institutional level, among the top 20 most productive institutions, China holds eight positions, visually reflecting China’s rapid development and outstanding achievements in ER research over the past decade.

From 2000 to May 31, 2024, the scientific literature on ER was widely disseminated across various journals, with *Fertility and Sterility* standing out in the Q1 quartile with an IF of 6.6, publishing 267 core articles, accounting for 7.96% of the total publications, showcasing its leading role in this research direction. The journal co-citation network analysis constructed using VOSviewer (as shown in Figure 3B) highlights *Fertility and Sterility*, *Biology of Reproduction*, and *Human Reproduction* as key information sources in the ER research field due to their high overall link strength, emphasizing their central role in knowledge dissemination and academic influence.

Regarding author contributions, Simon Carlos tops the list with 53 publications, demonstrating not only high productivity but also active promotion of interdisciplinary collaboration. Pellicer Antonio (36 publications) and Taylor Hugh S. (32 publications) follow closely, also making significant contributions to the field. Notably, Simon Carlos’s core research team (represented by the blue cluster) has achieved rich outcomes through in-depth and broad collaborative research, advancing the field significantly in various dimensions such as endometrial receptivity, related gene expression, leptin gene function, different assisted reproductive technologies, and the impact of various diseases on ER (21–25).

In the author co-citation network, Professor Lessey B.A., owing to his extensive research and profound insights in women’s health and ER, emerges as the most cited scholar. His work encompasses cutting-edge topics like the impact of ARID1A on the pregnant endometrium, SIRT1 and progesterone resistance mechanisms, and collaborates closely with top researchers like Simon Carlos and Pellicer Antonio. Additionally, he demonstrates expertise in other gynecological diseases such as endometriosis and polycystic ovary syndrome (26–30), further consolidating his authority in the field.

### 4.2 Hotspots analysis

Highly cited literature forms the cornerstone of knowledge in every research field (31), which not only elucidates the core issues of exploration but also lays a solid foundation for the discipline. The ten most frequently cited articles focus on four key areas:

(1) Precise Timing of Embryo Implantation: The optimal window for embryo implantation into the mother is 8–10 days after ovulation, with the successful implantation rate decreasing as time from ovulation increases (32).
(2) Molecular and Morphological Markers of ER: Ongoing exploration of molecular and morphological markers of ER plays a crucial role in the embryo implantation process (7, 17, 20, 33, 34).
(3) Dynamic Changes in ER Gene Expression: Studies on human endometrial biopsy reveal variation patterns in ER gene expression profiles across different menstrual cycle phases (19, 35).
(4) Personalized Embryo Transfer (PET) Strategies: With a deeper understanding of ER mechanisms, personalized embryo transfer strategies, particularly utilizing endometrial receptivity arrays (ERA), have emerged as significant advancements in addressing recurrent implantation failure (RIF) (36).

Furthermore, keyword clustering analysis identifies the primary subdomains within ER research. The resulting timeline view and time zone maps offer insights into the major branches and evolutionary trajectories of ER studies, showcasing remarkable progress from fundamental mechanism exploration to clinical application guidance.

Early ER research (prior to 2010) centered on the gene expression profile of the endometrium and the various hormone receptors and molecular markers within it. For instance, in 2002, it was found that during the WIO, 156 genes were significantly upregulated and 377 genes were significantly downregulated (37). Additionally, abnormal expression of endometrial markers, including estrogen receptors, LIF, and integrin, can impact receptivity (38, 39). Studies also explored the potential adverse effects of drug interventions on receptivity, such as the anti-estrogenic activity of omeprazole and the upregulation of endometrial apoptosis genes induced by ovarian stimulation (40, 41). However, ongoing research is dedicated to identifying ovulation-stimulating drugs that do not interfere with ER, like letrozole, which remains extensively employed in clinical settings (42).

Since 2006, the relationship between PCOS and ER has garnered significant attention. Prior to this, it was observed that PCOS patients experienced higher rates of infertility and post-pregnancy miscarriage compared to healthy women (43, 44). Subsequent in-depth research unveiled the potential disruption of endometrial homeostasis in PCOS patients on receptivity (45). Post-2010, impaired endometrial receptivity emerged as a critical bottleneck in enhancing the success rate of *in-vitro* fertilization, with research focusing on ovarian stimulation (46), autoimmune responses such as elevated thyroid peroxidase antibodies (Anti-TPO) (47), and epigenetic factors like MicroRNA-30d (48). These discoveries have laid a solid theoretical foundation for current research on RIF.

### 4.3 Research frontiers

This article delves into the recently emerged and widely discussed academia hotspots, particularly focusing on the research pertaining to ER, with a notable emphasis on RIF and the application of frozen embryo transfer (FET)technology, a topic that has gained significant momentum since 2020 (31). Amidst the remarkable advancements in IVF technology, the clinical and research communities continuously explore novel avenues to optimize IVF protocols, aiming to enhance patient outcomes.

In recent years, the debate over whether fresh embryo transfer should be performed directly in the same IVF cycle or whether all embryos should be frozen and delayed until the next cycle has intensified. In recent years, there has been a growing debate as to whether fresh embryo transfer should be carried out straight away or whether all embryos should be frozen and deferred for transfer to a later cycle. At the heart of this disagreement is the relative relationship between the timing of embryo transfer and the ovulation cycle. The fresh embryo transfer strategy advocates the transfer of fresh embryos in the cycle immediately following ovarian stimulation for egg retrieval, whereas frozen embryo transfer advocates the freezing and preservation of all embryos after egg retrieval to allow for transfer under more optimal physiological conditions (49).

Since the successful introduction of IVF technology in 1978, the traditional means of transfer has been fresh embryo transfer (50). However, in recent years, more and more scholars tend to support frozen embryo transfer, arguing that at the end of the ovarian stimulation cycle, estradiol and progesterone levels often reach a “super physiological” state, which may be detrimental to the receptivity of the endometrium and reduce the success rate of pregnancy (51). In addition, this strategy can effectively adjust the WIO (8, 52) and prevent the occurrence of ovarian hyperstimulation syndrome (OHSS) in patients with high responses to controlled ovarian stimulation (COS) (53). Thanks to the leap forward in cryopreservation technology, especially the advancement from slow freezing to vitrification, the survival rate of embryos after thawing has reached over 95% (54), making frozen embryo transfer a new option for clinical practice in many countries (55).

Multiple clinical randomized trials currently conducted have shown that the pregnancy rate and live birth rate of frozen embryo transfer are equivalent to those of fresh embryo transfer (50, 56). However, for specific patient groups, such as PCOS patients, studies have found that selective frozen embryo transfer can significantly increase the live birth rate and reduce the risk of OHSS and pregnancy complications (57). Furthermore, embryo cryopreservation transfer shows obvious advantages for patients with more than 15 retrieved eggs, though the effect is limited for patients with fewer retrieved eggs (58). Patient acceptance of a freeze-all strategy is high, especially when it improves expected success rates or reduces neonatal complications (59). Clinicians, therefore, need to weigh the pros and cons and choose the most appropriate transplant strategy based on the patient’s specific situation to ensure the safety and effectiveness of the treatment.

Despite extensive research into embryo and endometrial factors, the overall success rate of assisted reproductive technology remains below 30% (60), with RIF posing a significant obstacle to progress (61). RIF is defined as the failure to achieve pregnancy after at least three IVF cycles with high-quality embryos in women under 40 years old, with approximately half of these cases remaining unexplained (62), further exacerbating subsequent success rates. Researchers have proposed various potential mechanisms for RIF, including uterine abnormalities, infections, metabolic and hormonal disorders, and immunological factors (63), among which endometrial immune dysregulation is widely recognized as a crucial contributor to decreased receptivity (61, 64).

To address this challenge, endometrial immune analysis has emerged as an innovative approach, serving as a vital basis for the formulation of personalized IVF/ICSI treatment plans. By tailoring treatments based on immune profiles, patients have experienced a notable increase in live birth rates post-embryo transfer, with recovery rates reaching approximately 40% (65). While some literature advises against routine use of receptivity tests to guide transfer timing in conventional IVF settings (excluding RIF and recurrent miscarriage patients) (11), customized embryo transfer strategies for RIF patients have proven effective in enhancing pregnancy and live birth rates (66).

In summary, as in vitro fertilization technology advances and research into ER mechanisms deepens, clinicians must precisely assess patients’ conditions and tailor treatment plans to enhance the success rate of IVF and ensure maternal safety. Future research directions should range from continuing to explore the mechanisms of ER decline in patients with repeated implantation failures to conducting clinical retrospective studies to improve the overall ART success and live birth rates to help more infertile patients.

### 4.4 Advantages and limitations

This study demonstrates the research successes in the field of ER research from 2000 to the present, including the underlying molecular mechanisms of ER and their translational applications in clinical practice. These studies have manifestation the critical role of the endometrium in embryo implantation and have provided scientific ideas and potential therapeutic avenues to address key problems in ART, particularly repeated implantation failure. With a better understanding of ER mechanisms, researchers can now use a variety of tools to assess individual patients to develop treatment plans that will improve ART success rates.

Furthermore, our study, by elucidating the most productive authors and institutions in the field of ER, can facilitate collaboration and communication among researchers. These exchanges can harness collective intelligence to tackle research challenges and propel the in-depth development of the ER field. Additionally, our research findings also explore the frontiers and hotspots of ER research, providing a reference for future research directions.

However, there are some limitations to this study. First, the literature analyzed was selected from the WoSCC database and did not include other databases such as Google Scholar. The singularity of data sources may limit the comprehensiveness and representativeness of the study results. Future studies need to further expand the data sources to more comprehensively reflect the current state of research in the field of ER. In addition, being limited to English-language articles may overlook research findings from non-English-speaking countries. This language bias may affect the overall understanding and assessment of endometrial fertility research globally, and future studies need to be more linguistically inclusive and open.

Another aspect that cannot be overlooked is that the number of citations a document receives is heavily influenced by time. Newly published research findings often struggle to match the citation counts of previously published papers within a short period. This phenomenon implies that even if some papers have groundbreaking or profound impacts in the academic field, they may not receive widespread citations commensurate with their value due to their relatively short publication history. Lastly, the software tools used in literature analysis are also variables that affect the accuracy of results. Different software platforms differ in data processing, algorithmic logic, and other aspects, leading to subtle differences in the final analysis outcomes.

## 5 Conclusion

This study employs bibliometric analysis to offer researchers in the field of ER a comprehensive perspective. Since 2000, there has been a remarkable surge in the number of publications in the ER research field, underscoring its vigorous growth. These studies primarily concentrate on delving into the pathophysiological mechanisms of ER, with the primary objective of enhancing clinical pregnancy rates and live birth rates, ultimately benefiting more infertile patients. Currently, addressing the ER issues in patients with recurrent implantation failure represents the forefront of research. The primary treatment approaches currently in use involve optimizing embryo transfer timing and employing innovative strategies such as immunotherapy. These cutting-edge analyses not only provide new insights into the treatment of ER but also offer researchers fresh research directions, assisting them in selecting journals for publishing their research findings, identifying potential collaborators, and staying abreast of the latest trends and advancements in the field.

## Footnotes

1
https://www.vosviewer.com
