# Supplementary material for: Global research trends in endometrial receptivity from 2000 to 2024: bibliometric analysis
Source: Front Med (Lausanne). 2024 Oct 30;11:1465893. doi: 10.3389/fmed.2024.1465893 (PMC11558532; doi:10.3389/fmed.2024.1465893)
Supplement: Supplementary file 1 [file Table_1.docx]

Cluster Summary

| **Cluster ID** | Size | **Silhouette** | Year | Cluster labels | Keywords obtained by LLR algorithm |
| --- | --- | --- | --- | --- | --- |
| #0 | 330 | 0.605 | 2009 | embryo implantation | embryo implantation;  *in vitro* fertilization; uterus; frozenembryo transfer;decidualization |
| #1 | 261 | 0.724 | 2009 | frozen embryo transfer | frozen embryo transfer;  ivf;endometrial thickness;  *in vitro* fertilization; fresh embryo transfer |
| #2 | 141 | 0.662 | 2008 | integrins | integrins;  uterine receptivity;  unexplained infertility;  pinopodes ;progesterone receptors |
| #3 | 130 | 0.728 | 2013 | recurrent implantation failure | recurrent implantation failure;  chronic endometritis; personalized embryo transfer;  endometrial scratching; repeated implantation failure |
| #4 | 75 | 0.764 | 2018 | intrauterine adhesions | Intrauterine adhesions;  Endometrial regeneration;  asherman syndrome; |
| #5 | 20 | 0.939 | 2012 | polycystic ovary syndrome | polycystic ovary syndrome; insulinresistance;  pcos;insulin;metformin |
| #6 | 15 | 0.978 | 2006 | levonorgestrel | Levonorgestrel;mifepristone;emergency contraception;postcoital contraception;ulipristal acetate |
| #7 | 13 | 0.965 | 2010 | alpha (v) beta (3) integrin | alpha(v) beta (3) integrin;  ovine uterus;  endometrial gene expression; growth factor ii;  interferon gamma |
| #8 | 8 | 0.977 | 2012 | plasma membrane  transformation | Plasma membrane transformation;  molecular determinants;  rho gtpases;erm protein; actin cytoskeleton |
| #9 | 6 | 0.997 | 2001 | aniline hydroxylase | aniline hydroxylase; dlormeloxifene;  aminopyrinen demethylas;  therapeutic agents;  estrogen agonistic |
